# Low uptake of malaria testing within 24 h of fever despite appropriate health-seeking among migrants in Myanmar: a mixed-methods study

**DOI:** 10.1186/s12936-018-2546-4

**Published:** 2018-10-29

**Authors:** Kyaw Thu Hein, Thae Maung Maung, Kyaw Ko Ko Htet, Hemant Deepak Shewade, Jaya Prasad Tripathy, Swai Mon Oo, Zaw Lin, Aung Thi

**Affiliations:** 1 Department of Medical Research, Ministry of Health and Sports, Yangon, Myanmar; 2 International Union Against Tuberculosis and Lung Disease (The Union), South-East Asia Office, New Delhi, India; 3 International Union Against Tuberculosis and Lung Disease (The Union), Paris, France; 4 Population Services International, Yangon, Myanmar; 5 Vector Borne Disease Control Program, Ministry of Health and Sports, Nay Pyi Taw, Myanmar; 6 National Malaria Control Program, Ministry of Health and Sports, Nay Pyi Taw, Myanmar

**Keywords:** Malaria diagnosis and treatment, Knowledge, health-seeking, Barriers, Suggestion, Uptake of malaria testing, Myanmar, SORT IT

## Abstract

**Background:**

There is limited information on uptake of malaria testing among migrants who are a ‘high-risk’ population for malaria. This was an explanatory mixed-methods study. The quantitative component (a cross sectional analytical study-nation-wide migrant malaria survey in 2016) assessed the knowledge; health-seeking; and testing within 24 h of fever and its associated factors. The qualitative component (descriptive design) explored the perspectives of migrants and health care providers [including village health volunteers (VHV)] into the barriers and suggested solutions to increase testing within 24 h. Quantitative data analysis was weighted for the three-stage sampling design of the survey. Qualitative data analysis involved manual descriptive thematic analysis.

**Results:**

A total of 3230 households were included in the survey. The mean knowledge score (maximum score 11) for malaria was 5.2 (0.95 CI 5.1, 5.3). The source of information about malaria was 80% from public health facility staff and 21% from VHV. Among 11 193 household members, 964 (8.6%) had fever in last 3 months. Health-seeking was appropriate for fever in 76% (0.95 CI 73, 79); however, only 7% (0.95 CI 5, 9) first visited a VHV while 19% (0.95 CI 16, 22) had self-medication. Of 964, 220 (23%, 0.95 CI 20, 26) underwent malaria blood testing within 24 h. Stable migrants, high knowledge score and appropriate health-seeking were associated with testing within 24 h. Qualitative findings showed that low testing within 24 h despite appropriate health-seeking was due to lack of awareness among migrants regarding diagnosis services offered by VHV, delayed health-seeking at public health facilities and not all cases of fever being tested by VHV and health staff. Providing appropriate behaviour change communication for migrants related to malaria, provider’s acceptance for malaria testing for all fever cases and mobile peer volunteer under supervision were suggested to overcome above barriers.

**Conclusions:**

Providers were not testing all migrant patients with fever for malaria. Low uptake within 24 h was also due to poor utilization of services offered by VHV. The programme should seriously consider addressing these barriers and implementing the recommendations if Myanmar is to eliminate malaria by 2030.

**Electronic supplementary material:**

The online version of this article (10.1186/s12936-018-2546-4) contains supplementary material, which is available to authorized users.

## Background

To decrease the risk of severe complications and onward transmission of malaria, World Health Organization has emphasized early diagnosis and prompt treatment within 24–48 h of onset of symptoms [1]. The advent and scale up of rapid diagnostic test kits has made early and accurate diagnosis of malaria possible at primary health care and community settings [1, 2].

Myanmar is one of the five countries in south-east Asia (Cambodia, Laos, Thailand, Vietnam, and Myanmar) with artemisinin resistance [3]. Myanmar reported more than 152 thousand confirmed cases in 2015, accounting over 70% of total cases in the greater Mekong sub-region [3, 4]. It has high malaria morbidity and mortality rates, third highest in the south-east Asia region and highest in the greater Mekong sub-region [5].

With the countrywide scale-up of malaria prevention and control measures under the National malaria control programme (NMCP), the malaria morbidity significantly reduced from 11.2 to 4.1 cases per 1000 population and malaria deaths reduced from 1707 to 92 between 2005 and 2014, respectively [4].

Malaria is now largely concentrated among hard-to-reach and other ‘populations at risk’, including internal migrants (henceforth called as migrants) who also work as forest goers and outdoor night-time workers [4, 5]. Ensuring universal access to malaria diagnosis and treatment among migrants is central to eliminating malaria by 2030 and Myanmar is no exception [4]. To eliminate malaria from the greater Mekong sub-region and prevent the spread of artemisinin drug resistant malaria, regional artemisinin-resistance initiative (RAI) project was launched in Myanmar with the support of the Global Fund in 2014. One of the objectives was to provide access to prevention, diagnosis and treatment for migrants [6, 7].

Previously, there have been studies on awareness of malaria, health-seeking behaviour and testing within 24 h of fever among general population in the endemic regions of Myanmar and treatment-seeking preferences within the public sector amongst migrant workers. However malaria testing among migrants has not been reported yet [8–10].

A nation-wide migrant malaria survey was conducted jointly by the department of medical research (DMR) and NMCP in 2016 to understand the knowledge, attitude and health-seeking behaviour towards malaria and the ownership and utilization of insecticide-treated bed nets among the migrant population in the selected townships of RAI supported areas. This provides us a unique opportunity to study the uptake of malaria testing (within 24 h) among migrant population and the associated factors. Secondly, a systematic qualitative enquiry into the perspective of migrants and health care providers including village health volunteers (VHV) into barriers to access malaria testing despite availability of rapid diagnostic test kits at village level would aid the programme in designing specific interventions or modify existing intervention to suit the needs of migrants.

Hence, this mixed-methods study was conducted to assess the knowledge; health-seeking; and malaria testing within 24 h of fever and its barriers among migrant population in RAI supported areas in Myanmar.

## Methods

### Study design

This was an explanatory mixed-methods study involving a quantitative component (a cross sectional analytical study-nation-wide migrant malaria survey 2016) followed by a descriptive qualitative component where the findings from the quantitative component fed into the qualitative component [11].

### General study setting

In the south-east Asian region, the Republic of the Union of Myanmar is neighboured by countries like Bangladesh, India, China, Laos and Thailand in the North and East [5]. It is divided into seven states, seven regions and one capital territory (Nay Pyi Taw Council territory). There are 74 districts with 330 townships [12]. In 2014, 9.4 million people were internal migrants, which is approximately 20% of the total population. Myanmar has grown to be the largest migration source country in greater Mekong sub-region [13].

### Specific study setting

#### Health care services in Myanmar

Township hospitals provide health care services including laboratory, dental and also major surgical procedures and act as the first referral health institutions. Station hospitals including sub-township hospitals are basic medical units with essential curative elements, such as general medical, surgical and obstetric facilities. Basic health staff (BHS) are the major community-based health workforce responsible for providing comprehensive health care services and are stationed at rural health centre (RHC) or the sub-centres under RHCs. RHC is staffed by health assistant, one lady health visitor, five midwives and five public health supervisors. Sub-centre is staffed by one midwife and one public health supervisor. VHVs work under the supervision of midwife at sub-centre [12].

#### National malaria control programme (NMCP)

In Myanmar, 291 out of 330 townships are malaria endemic with about 44 million at risk of the disease. All cases of fever should undergo a rapid diagnostic test (SD-Bioline malaria Ag P.f/P.v combo 25) within 24 h for detection of *Plasmodium falciparum* and/or non-*falciparum* infections. It is available at all public health facilities including township/station hospitals and RHCs. The same has also been decentralized at the level of BHS and VHV. All malaria diagnostic and treatment services are offered free of cost [14].

#### RAI supported area

Of the 291 endemic townships, 52 were covered under RAI project in 2015 and 76 were covered in 2016. In RAI areas, migrant clusters were divided into three categories: permanent or semi-permanent work settings with high social capital, where substantial results can be achieved for malaria control (category I); semi-permanent settings with moderate social capital, where substantial community-based results can be achieved for malaria control (category II); and small, often temporary work sites, with low social capital and resource availability (category III) [8].

### Study population

#### Quantitative component

The migrant populations covered in the nation-wide migrant malaria survey 2016 (August to December) were included. A migrant was a male or female of any age who temporarily lived in the selected townships for less than 3 years duration of stay and not registered as a native villager in the village census.

The planning for the nation-wide migrant malaria survey 2016 was done based on the 2015 data profile. As 52 townships were part of the RAI areas in 2015, it was decided in 2015 to implement the 2016 survey in these 52 townships.

**Fig. 1:**
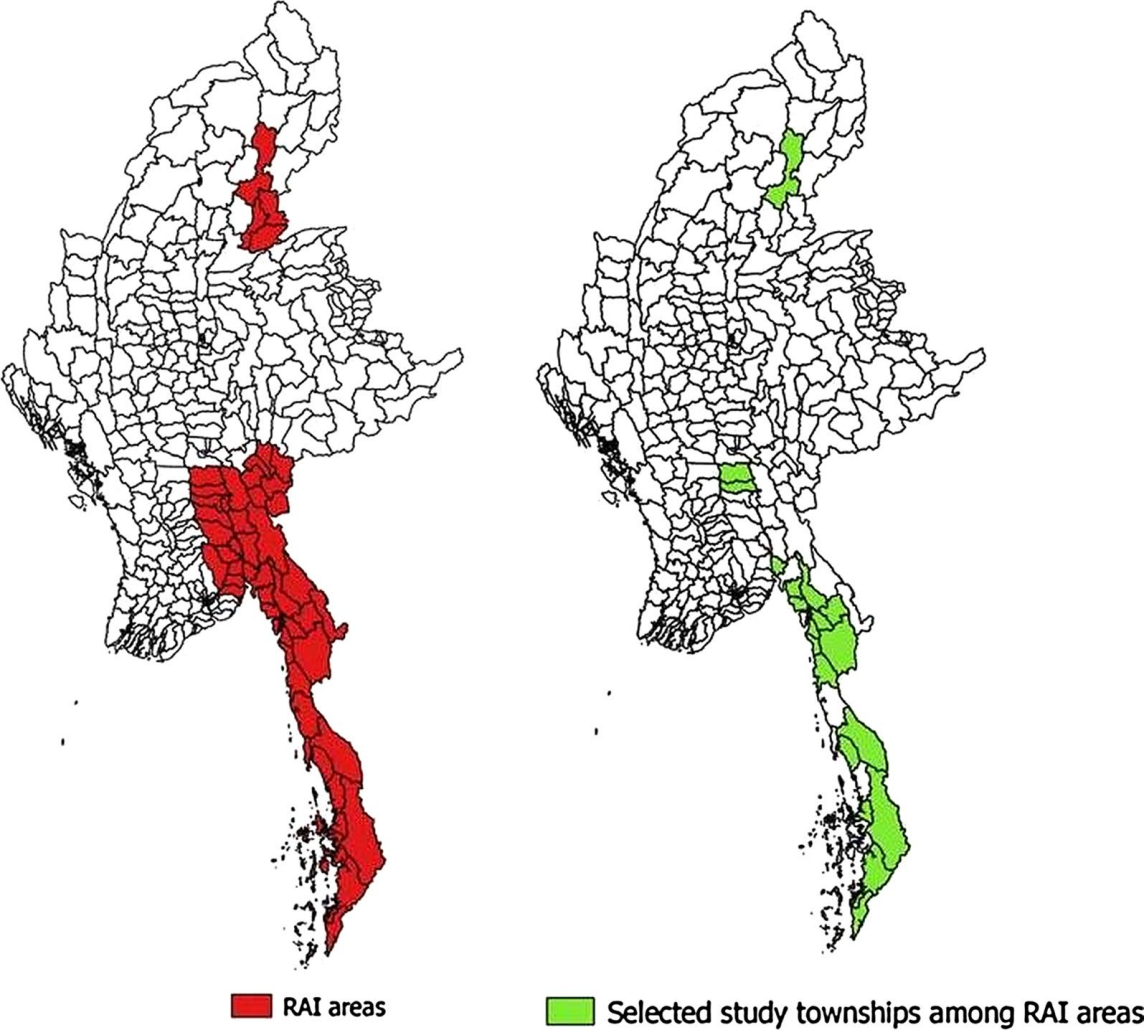
Map of Myanmar showing regional artemisinin-resistance initiative (RAI) townships and selected RAI townships for nation-wide migrant malaria survey in 2016

A multistage sampling procedure was employed. Out of 52 townships in RAI areas, 13 townships were excluded as they were geographically hard-to-reach. Among 39 townships, 27 were selected by probability proportional to size **(**Fig. 1). In each selected township, migrant clusters were mapped and five migrant clusters were randomly (simple random sampling) selected. A list of households in these five clusters was prepared. A total of 125 households were selected from 5 clusters in each township and the number of households sampled from each cluster was proportional to the total number of households in that migrant cluster.

#### Qualitative component

Two state/regions (Bago and Sagaing) that contained the RAI areas and had large sites of migrant population with wide variations in occupational characteristic were purposively selected. In each state/region, two RAI townships that were part of the nation-wide migrant malaria survey 2016 were selected (Taungoo and Yedashe Townships in Bago, Homalin and Kalay Townships in Sagaing).

Programmatic challenges in early diagnosis and treatment of malaria among migrants and their suggested solutions were explored. Provider side (VHV, BHS, local vector-borne disease control staff, regional medical officer) and client side participants (migrant people of varying occupation with or without history of fever in last 3 months) were selected based on their availability at the time of visits by the research team—December 2017 to January 2018 (convenience sampling).

### Data collection

#### Quantitative component

Under migrant malaria survey 2016, face-to-face interview was conducted with preferably the female adult respondent or any other adult using a semi-structured questionnaire by a trained interviewer (see Additional file 1).

Questionnaires were pre-tested and all interviewers were well trained in each state/region by NMCP and DMR. The questionnaires included household characteristics, household level knowledge regarding malaria (cause, transmission, risk groups, signs/symptoms, prevention and treatment) and household level sources of information. Among individuals in the household who had fever in last 3 months, health-seeking for fever and testing for malaria diagnosis within 24 h was assessed.

#### Qualitative component

One-to-one interviews among key informants and focus group discussion-FGD among migrant people were conducted. Two researcher officers from DMR who were medical doctors conducted the qualitative enquiry. They were trained in qualitative research. Though they were not part of NMCP, they were knowledgeable and understood the implementation of NMCP and the RAI project.

One-to-one interviews and FGDs were arranged at the place convenient to participants: health facility or residence for key informants, work sites for migrants. Participants were informed the purpose of the study before the visit. Interview and FGD guides were pilot-tested before implementation in field **(**see Additional files 2 and 3). Audio recording (after consent) and verbatim notes were taken during the interview. After the interview/FGD was over, the summary was read back to the participants to ensure participant validation. Field notes (if any) from observations during data collection were also made.

### Data management and analysis

#### Quantitative component

Survey data was double-entered and validated using EpiData entry software (version 3.1, EpiData Association, Odense, Denmark) and analysed using STATA (version 12.1 STATA Corp., College Station, TX, USA).

The household and individual characteristics were described by using means (95% confidence interval (CI)) for continuous variables and frequencies and proportions (95% CI) for categorical variables. Questions related to knowledge about malaria—its cause, transmission, signs and symptoms, vulnerable groups, severity, prevention were scored as 1 (yes) and 0 (no). Specific scores were summed up to get overall knowledge score for each individual in the survey **(**see Additional file 4). The maximum and minimum scores were 0 and 11. The score were then categorized into high (> 5) and low (≤ 5) based on the mean cut-off value. Health-seeking was considered ‘appropriate’ if the visit was made to a VHV or any public health facility or private clinics of doctor/health assistant/auxiliary midwife.

The factors associated with malaria testing within 24 h of fever were determined using multivariable log-binomial regression analysis. Variables having a *p*-value less than 0.2 in unadjusted analysis were included. Prevalence ratio (unadjusted and adjusted) along with 95% CI were calculated.

The quantitative analysis was weighted for the multi-stage sampling design and hence, weighed estimates have been provided [15]. The probability of selection of townships and selection of the migrant cluster were used to derive the weights. Information on probability of selection of households within clusters was not available and not included in the weights.

#### Qualitative component

The transcripts were made based on the verbatim notes and listening to the audio records. The transcripts obtained were compiled and the principal investigator (KTH) read the transcripts to become familiar with the data. Manual descriptive thematic analysis was used to analyze the transcripts [11, 16]. It was reviewed by a second investigator (KKKH) to reduce bias and interpretive credibility. The decision on coding rules and theme generation was done by using standard procedures and in consensus [17]. Any differences between the two were resolved by discussion. This approach in health-care research is flexible and appropriate for determining solutions to real-world problems. Both inductive and deductive codes were generated. Similar codes were combined into themes [11]. The codes/themes were related back to the original data to ensure that the results are a reflection of the data [18]. The themes were and relevant quotes were summarized in a table. The findings were reported by using ‘Consolidated Criteria for Reporting Qualitative Research’ [19].

## Results

### Quantitative findings

#### Migrant household characteristics

A total of 3230 households were included and their characteristics have been summarized in Table 1. The mean household size was 3.4, 40% (n = 1294) stayed in temporary shelters made by themselves, 39% (n = 1251) belonged to migrant category III.

**Table 1:** Background characteristics of the migrant households in regional artemisinin resistance initiative (RAI) areas of Myanmar, 2016 [N = 3230]

Variables	N	Proportion^a^	(95% CI)
Total	3230	100	–
House hold size			
1–2	1153	35.7	(33.8, 37.5)
3–4	1254	38.8	(36.9, 40.7)
5–6	600	18.5	(17.1, 20.1)
>6	223	6.9	(5.9, 7.8)
Mean	3230	3.4	(3.3, 3.5)
Housing status			
Own house	1294	40.1	(38.2, 41.9)
Rental	40	1.2	(0.8, 1.6)
Employer allowed place	1624	50.3	(48.3, 52.2)
Not known	272	8.4	(7.2, 9.7)
Main occupation of family			
Farming/gardening/rubber plantation work	1231	38.1	(36.2, 40.0)
Stone mining work/brick kiln work	817	25.3	(23.5, 27.0)
Merchant	38	1.2	(0.8, 1.5)
Daily wage labourer	572	17.7	(16.3, 19.2)
Not known	572	17.7	(16.2, 19.2)
Migrant category^b^			
Category I	756	23.4	(21.8, 24.9)
Category II	982	30.4	(28.6, 32.2)
Category III	1251	38.7	(36.8, 40.6)
Not known	241	7.5	(6.3, 8.6)
Duration of intention to stay			
Less than 6 months	415	12.8	(11.4, 14.3)
6 months to 1 year	204	6.3	(5.3, 7.3)
More than 1 year	366	11.3	(10.2, 12.5)
Not known	2245	69.5	(67.7, 71.4)
Location of intent to go back			
Native place	1263	39.1	(37.2, 41.0)
Another workplace within township	195	6.1	(5.2, 6.9)
Another workplace another township	72	2.2	(1.7, 2.8)
Not known	1700	52.6	(50.7, 54.6)
Knowledge score			
Mean	3230	5.2	(5.1, 5.3)

Weighted estimates given taking into account the sampling design

*CI* confidence interval

^a^Column percentages

^b^Permanent or semi-permanent work settings with high social capital, where substantial results can be achieved for malaria control (category I); semi-permanent settings with moderate social capital, where substantial community-based results can be achieved for malaria control (category II); and small, often temporary work sites, with low social capital and resource availability (category III)

#### Knowledge regarding malaria among migrant households

**Fig. 2:**
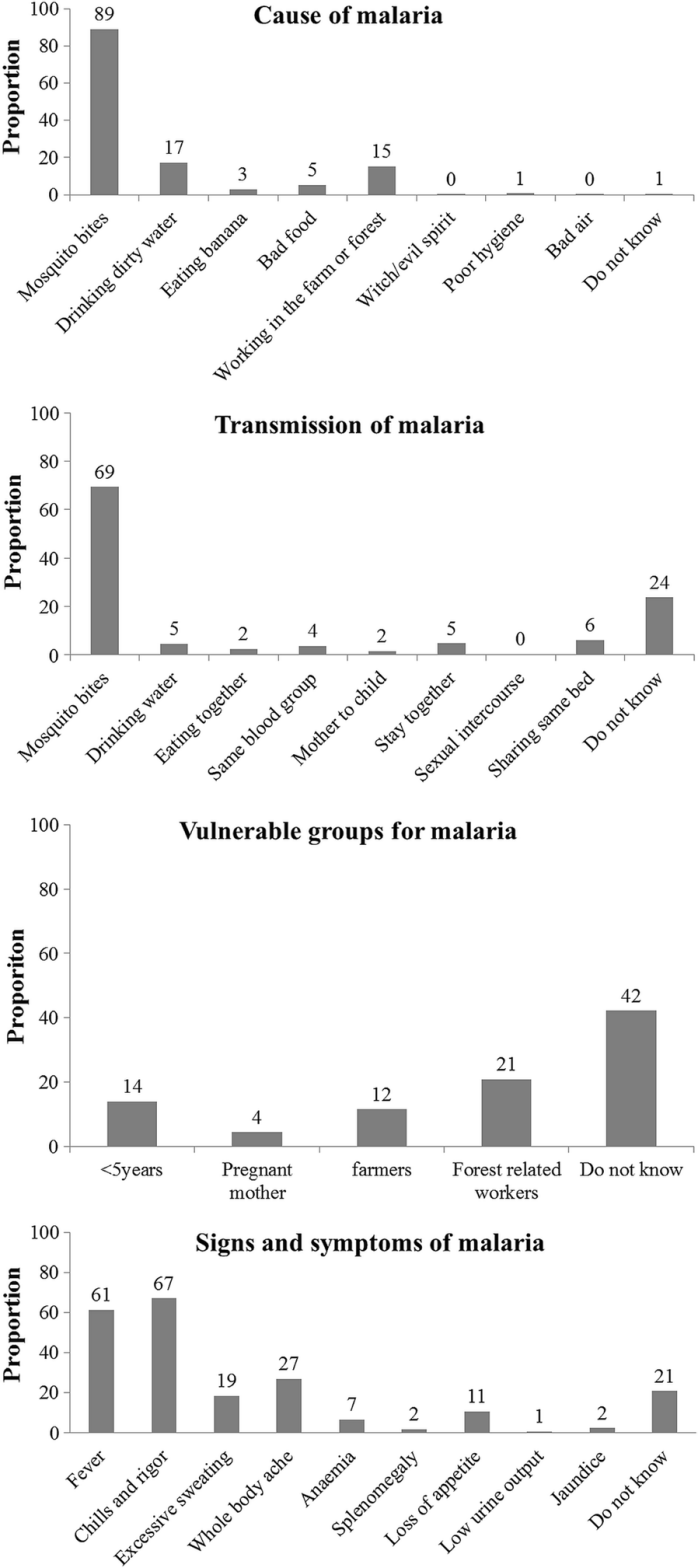
Knowledge regarding cause, transmission, vulnerable group and signs and symptoms of malaria among migrant households in regional artemisinin-resistance initiative (RAI) areas of Myanmar, 2016 [N = 3230]*. *Weighted estimates given taking into account the sampling design. *Multiple responses possible

**Fig. 3:**
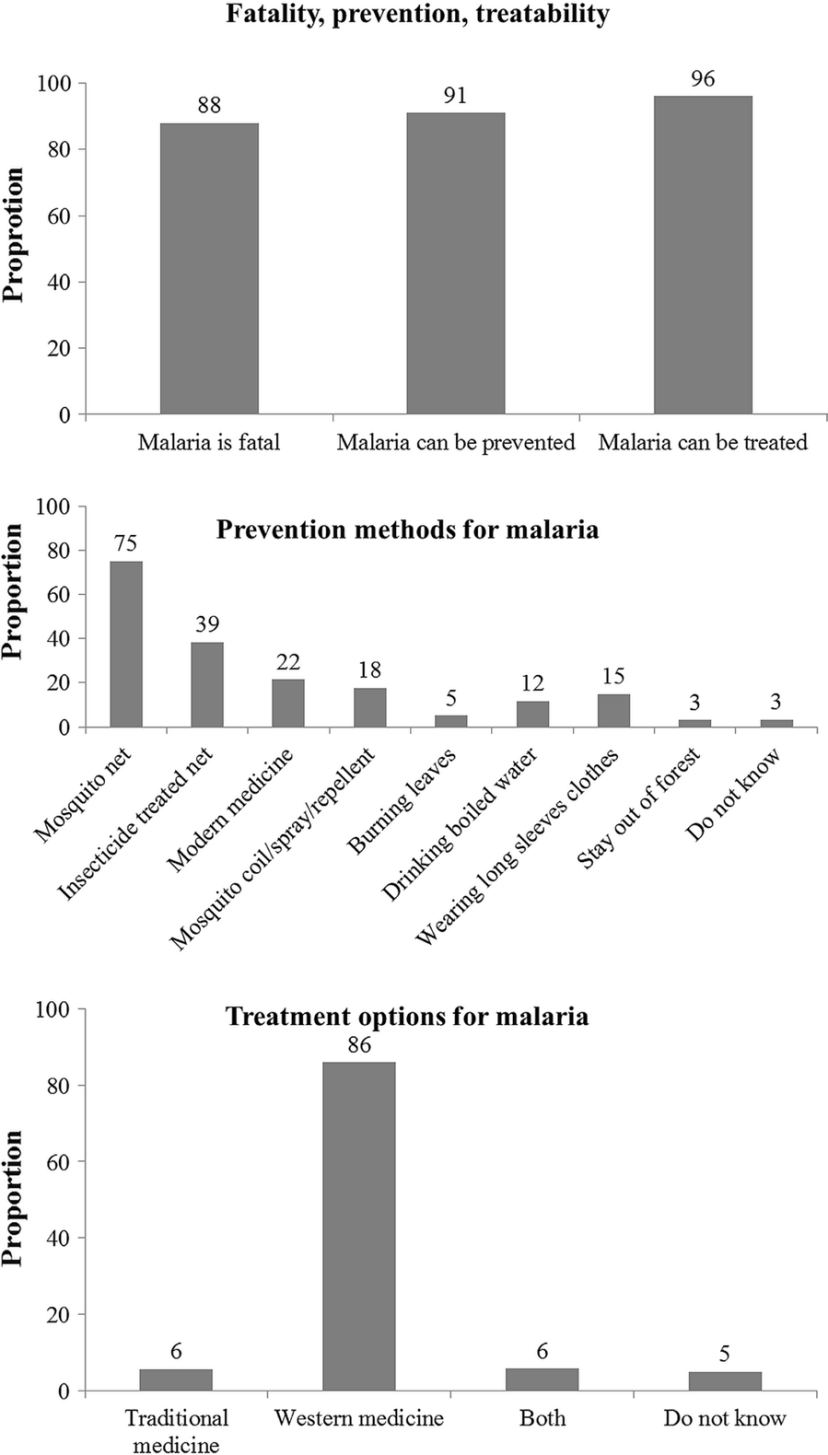
Knowledge regarding prevention and treatment of malaria among migrant households in regional artemisinin-resistance initiative (RAI) areas of Myanmar, 2016 [N = 3230]*. *Weighted estimates given taking into account the sampling design. *Multiple responses possible

The mean (0.95 CI) knowledge score (maximum score 11) among households was 5.2 (5.1, 5.3) (Table 1). The knowledge on cause, transmission, vulnerable groups, symptoms, prevention and treatment regarding malaria has been summarized in Figs. 2, 3. Though 89% knew that malaria is caused by mosquito bites, 31% did not mention mosquitoes as a source of transmission. Fourteen percent recognized under-five children as a vulnerable group for malaria, while 42% did not know anything about vulnerable groups. Fever and chills/rigors were mentioned as symptoms of malaria by 61% and 67% household, respectively. Malaria was preventable and treatable according to 91% and 96% household, respectively. Western medicine was identified as a treatment option for malaria by 86% of the households.

#### Sources of information regarding malaria among migrant households

**Fig. 4:**
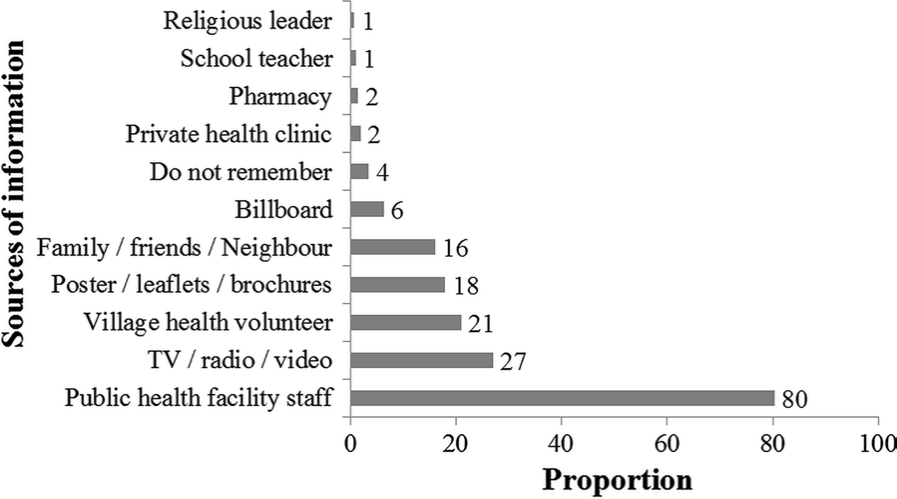
Sources of information for knowledge regarding malaria among migrant households in regional artemisinin-resistance initiative (RAI) areas of Myanmar, 2016 [N = 3230]*. *Weighted estimates given taking into account the sampling design. *Multiple responses possible

The sources of information regarding malaria are depicted in Fig. 4. Public health facility staff and VHV were the sources for 80% and 21% households, respectively.

#### Migrants with fever: characteristics and health-seeking

Of 11,193 household members, 964 (8.6%) had fever in last 3 months. The individual, household and health-seeking characteristics of these 964 individuals have been summarized in Table 2. Mean age was 24.6 years and 55% were males. After the onset of fever, health care was first sought from the sub-centre/RHC, township/station hospital, private clinic of midwife or health assistant and private clinic of doctor by 39%, 10%, 9%, and 7%, respectively. Seven percent first visited a VHV while self-medication was done by 19%. There was a question on ‘difficulty to visit a VHV’ in the migrant malaria survey; however, data was missing for majority.

**Table 2:** Background characteristics andhealth-seeking for malaria among migrants with fever in last 3 months in regional artemisinin-resistance initiative (RAI) areas of Myanmar, 2016 [N = 964]

Variables	N	Proportion^a^	(95% CI)
Total	964	100	–
Individual level			
Age n years			
< 5	175	18.2	(15.5, 20.9)
5–14	196	20.3	(17.4, 23.1)
15–59	538	55.8	(52.2, 59.4)
≥ 60	22	2.3	(1.2, 3.4)
Not known	33	3.4	(1.9, 4.9)
Mean	964	24.6	(22.9, 26.3)
Sex			
Male	526	54.5	(50.9, 58.2)
Female	438	45.5	(41.8, 49.1)
Household level			
House hold size			
1–2	149	15.5	(12.7, 18.3)
3–4	372	38.6	(35.0, 42.1)
5–6	332	34.5	(31.0, 37.9)
> 6	111	11.5	(9.2, 13.8)
Mean	964	4.4	(4.2, 4.5)
Housing status			
Own house	433	44.9	(41.3, 48.5)
Rental	10	1.0	(0.3, 1.7)
Employer allowed place	467	48.5	(44.8, 52.1)
Not known	54	5.6	(3.7, 7.6)
Main occupation of family			
Farming/gardening/rubber plantation work	285	29.6	(26.2, 33.0)
Stone mining work/brick kiln work	236	24.4	(21.3, 27.6)
Merchant	20	2.1	(1.2, 3.0)
Daily wage labourer	212	22.0	(19.0, 25.0)
Not known	212	22.0	(19.0, 25.0)
Migrant category^b^			
Category I	329	34.1	(30.6, 37.6)
Category II	222	23.0	(19.9, 26.0)
Category III	369	38.3	(34.6, 41.8)
Not known	45	4.7	(2.8, 6.5)
Duration of intention to stay			
Less than 6 months	92	9.6	(7.3, 11.9)
6 months to 1 year	81	8.4	(6.2, 10.5)
More than 1 year	167	17.3	14.7, 19.9)
Not known	624	64.8	(61.3, 68.3)
Knowledge score^c^			
Mean	964	5.1	(4.9, 5.3)
Health-seeking			
Inappropriate	209	21.7	(18.6, 24.7)
No medication	16	1.7	(0.6, 2.7)
Self-medication	186	19.3	(16.4, 22.2)
Quack	7	0.7	(0.1, 1.3)
Appropriate	734	76.1	(73.0, 79.2)
Trained VHV	67	6.9	(5.0, 8.9)
RHC/sub-centre	380	39.4	(35.8, 42.9)
Township/station hospital	95	9.9	(7.7, 12.1)
Private clinic	68	7.0	(5.2, 8.8)
Private clinic of midwife/HA	88	9.1	(6.9, 11.3)
AMW	36	3.8	(2.3, 5.3)
Not known	21	2.2	(1.3, 3.1)

Weighted estimates given taking into account the sampling design

*VHV* village health volunteer, *RHC* rural health centre, *HA* health assistant, *AMW* auxiliary midwife; *CI* confidence interval

^a^Column percentages

^b^Permanent or semi-permanent work settings with high social capital, where substantial results can be achieved for malaria control (category I); semi-permanent settings with moderate social capital, where substantial community-based results can be achieved for malaria control (category II); and small, often temporary work sites, with low social capital and resource availability (category III)

^c^Maximum knowledge score is 11

#### Migrants with fever: testing for malaria within 24 h

**Fig. 5:**
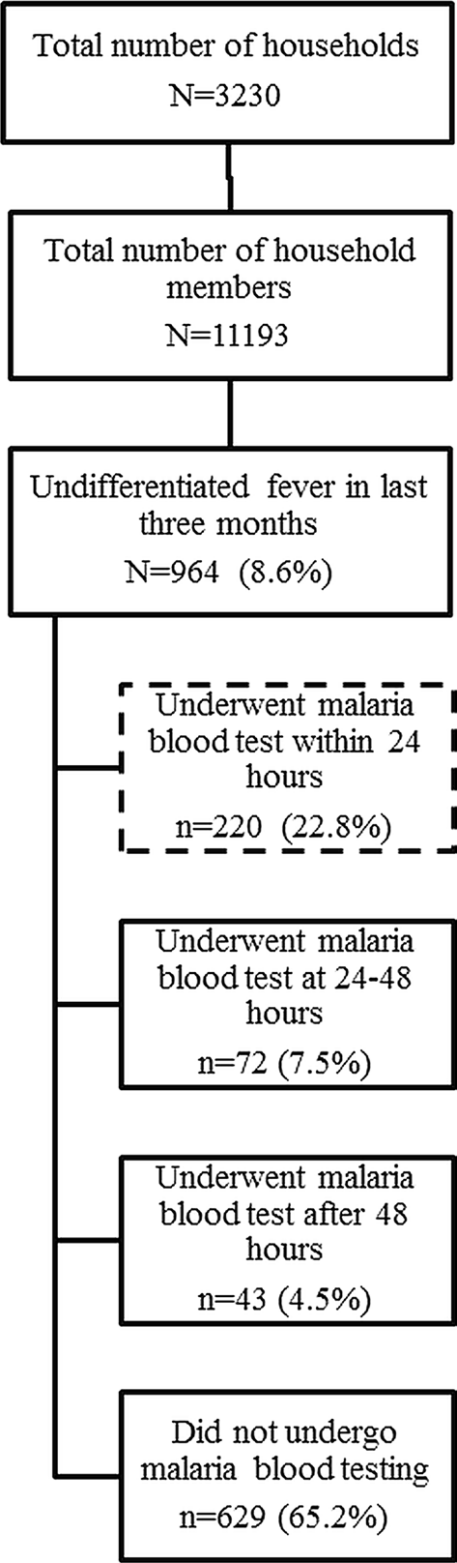
Flow diagram depicting the malaria testing among migrants with fever in last 3 months in regional artemisinin-resistance initiative (RAI) areas of Myanmar, 2016 [N = 964]*. *Weighted estimates given taking into account the sampling design. Of 964, 734 (76.1%) sought appropriate care, 347 (36.0%) were tested, malaria was diagnosed in 13.5% (47/347)

Of 964, 734 (76.1%) sought appropriate care and 347 (36.0%) underwent malaria blood testing. Among 347, malaria was diagnosed in 47 (13.5%). Testing within 24 h among migrants with fever was seen in 220 [22.8% (0.95 CI: 19.9%, 25.8%)] (Fig. 5). Factors associated with testing within 24 h have been shown in Table 3. The factors were: age 5–59 years, migrant category I, long (> 1 year) intention to stay, high (> 5) knowledge score and appropriate health-seeking.

**Table 3:** Association of background characteristics and health-seeking with malaria testing within 24 h of fever among migrants in regional artemisinin-resistance initiative (RAI) areas of Myanmar, 2016 [N = 964]

Variables	Total	Test within 24 h	PR (95% CI)	aPR (95% CI)
		N (%)^a^		
Total	964	220 (22.8)		
Individual level				
Age n years				
< 5	175	31 (17.8)	Ref	Ref
5–14	196	53 (27.1)	1.51 (0.99, 2.31)	1.51 (1.01, 2.25)^b^
15–59	538	127 (23.7)	1.32 (0.90, 1.92)	1.57 (1.09, 2.26)^b^
≥ 60	22	5 (21.2)	1.18 (0.44, 3.21)	1.49 (0.66, 3.36)
Not known	33	3 (10.5)	0.58 (0.17, 1.98)	0.98 (0.34, 2.81)
Sex				
Male	526	113 (21.5)	Ref	Ref
Female	438	107 (24.4)	1.14 (0.88, 1.47)	1.09 (0.86, 1.38)
House hold level				
House hold size				
1–2	149	20 (13.5)	Ref	Ref
3–4	372	81 (21.9)	1.62 (1.01, 2.61)	1.04 (0.65, 1.68)
5–6	332	97 (29.1)	2.16 (1.35, 3.47)	1.56 (0.98, 2.49)
>6	111	22 (19.8)	1.47 (0.80, 2.69)	1.22 (0.67, 2.20)
Housing status				
Own house	433	112 (25.9)	1.15 (0.89, 1.49)	–^c^
Rental	10	3 (29.6)	1.31 (0.47, 3.65)	–^c^
Employer allowed place	467	105 (22.5)	Ref	Ref
Not known	54	0 (0.0)	–	–
Main occupation of family				
Farming/garden/rubber	285	77 (26.9)	1.56 (1.04, 2.34)	1.20 (0.83,1.74)
Stone mining work/brick kiln	236	41 (17.3)	ref	ref
Merchant	20	3 (15.3)	0.88 (0.36, 2.19)	0.94 (0.35, 2.50)
Daily wage labourer	212	51 (24.1)	1.40 (0.91, 2.13)	1.07 (0.72, 1.59)
Not known	212	49 (22.9)	1.33 (0.86, 2.04)	1.28 (0.87, 1.89)
Migrant category^d^				
Category I	329	113 (34.3)	1.86 (1.39, 2.49)	1.83 (1.39, 2.41)^b^
Category II	222	39 (17.6)	0.95 (0.64, 1.42)	0.95 (0.65, 1.41)
Category III	369	68 (18.5)	Ref	Ref
Not known	45	0 (0.0)	–	–
Knowledge score^e^				
≤5	485	83 (17.0)	Ref	Ref
>5	479	138 (28.7)	1.69 (1.28, 2.22)	1.42 (1.09, 1.85)^b^
Duration of intention to stay				
Less than 6 month	92	7 (7.4)	Ref	Ref
6 month to 1 year	81	9 (10.8)	1.46 (0.51, 4.22)	1.37 (0.48, 3.91)
More than 1 year	167	36 (21.5)	2.90 (1.25, 6.74)	2.54 (1.07, 6.01)^b^
Not known	624	169 (27.0)	3.64 (1.63, 8.10)	3.27 (1.41, 7.54)^b^
Health seeking				
Inappropriate	209	13 (6.1)	Ref	Ref
Appropriate	734	204 (27.8)	4.56 (2.60, 8.00)	4.25 (2.41, 7.49)^b^
Not known	21	3 (15.8)	2.60 (0.96, 7.06)	2.54 (1.00, 6.42)^b^

All the estimates are weighted estimates taking into account the sampling design

*PR* prevalence ratio, *aPR* adjusted prevalence ratio (log binomial regression), *CI* confidence interval

^a^Row percentages

^b^p < 0.05

^c^Not included in the model as unadjusted p value for association with testing within 24 h was > 0.2

^d^Permanent or semi-permanent work settings with high social capital, where substantial results can be achieved for malaria control (category I); semi-permanent settings with moderate social capital, where substantial community-based results can be achieved for malaria control (category II); and small, often temporary work sites, with low social capital and resource availability (category III)

^e^Maximum knowledge score is 11

### Qualitative findings

A total of 17 one-to-one interviews and 17 FGDs (involving 121 migrants) were done. The characteristics of key informants and FGD participants have been summarized in Table 4. The perceived barriers and suggested solutions along with the relevant quotes have been summarized in Table 5.

**Table 4:** Socio-demographic details of the key informants involved in one-to-one interviews and migrants involved in focus group discussions (FGDs) in four regional artemisinin-resistance initiative (RAI) townships of Myanmar (Dec 2017–Jan 2018)

	FGD participants	Key informants
	N (%)	N (%)
Total	121 (100)	17 (100)
Gender		
Male	77 (64)	14 (82)
Female	44 (36)	3 (18)
Age group (years)		
15–24	26 (21)	1 (6)
25–44	67 (55)	9 (53)
45–64	27 (22)	6 (35)
65 years and above	1 (1)	1 (6)
Years of service (years)		
0–5	–	8 (47)
6–10	–	2 (12)
More than 10 years	–	5 (29)
Missing	–	2 (12)

FGD participants were bamboo cutters (n = 15), bridge construction workers (n = 15), charcoal makers (n = 11), fishermen (n = 13), gold miners (n = 16), oil diggers (n = 19), stone mine workers (n = 12), forest worker (n = 3), teak plantation workers (n = 6), others (n = 11)

Key informants were health assistant (n = 2), malaria assistant (n = 2), malaria supervisor (n = 3), public health supervisor (n = 2), village health volunteers (n = 8); the FGDs (n = 17) and one-to-one interviews (n = 17) were done in four townships namely Homalin, Kalay, Yay Tar Shay, Taungoo in two regions Bago and Sagaing

**Table 5:** Provider level and migrant level perceived barriers and suggested solutions to increase timely malaria testing among migrants in regional artemisinin-resistance initiative (RAI) areas of Myanmar (Dec 2017–Jan 2018)

Categories	Theme	Verbatim quotes
Migrant level	Inappropriate health care seeking	“*He* [Quack] *is specialized in malaria. We usually go to him when face with sickness* [fever]” (28 year old female stone mine worker)
	Self-medication	“*We will go for health care provider if we cannot stand because of fever*. [We take] *some traditional medicines for mild fever*” (49 year male fisherman)
	Not giving importance to fever	“*I have no idea to say for timely testing of every fever cases. Actually we sleep when having fever and when feel recovered, go to work.”* (36 year male construction worker) *“We will not go for health care and will go when not recovering from sickness”* (24 year male charcoal maker)
	Transportation difficulty	*“Going to the bottom of this mountain* [to rural health centre or village health volunteer] *is mainly difficult, especially in rainy season, the only possible way is walking.”* (52 year male oil digger)
	Not affordable	*“Even the blood testing is free of charges but needed to pay for the treatment for the fever.”* (30 year female charcoal maker)
	Previous experiences of no timely testing	*“The health care providers, here is midwives from public clinics, asking previous history of experiencing malaria and what medication taking when we go with fever. No experiences on malaria blood testing”* (45 year male fisherman)
	Uninformed about VHV or activities of VHV	“*Firstly, it* [malaria blood test] *can be done at Pauk Taw* [sub centre] *then also possible at Inn Din* [private clinic] *and finally we can go Kalay* [hospital or private clinic].” (28 year male oil digger) *“We didn’t know and not heard about malaria volunteer* [VHV]”. (39 year female oil digger, 36 year male oil digger and 51 year male oil digger)
	Lack of symptomatic treatment from a VHV	*“Only malaria diagnosis is not sufficient. There should be treatment for other symptoms as well”* (33 year male fisherman)
Provider level	Lack of practices for malaria testing	*“If the symptoms is as malaria, then giving blood test and not undergo blood test for the simple sickness* [fever]*.”* (34 year male BHS)
	Afraid of paper workload	*“Because VHV afraid to fill in the needed forms, some are not trying to do blood testing”* (39 year male vector borne disease control staff)
	Waste of test kits in no burden area	*“They* [BHS and VHV] *not try to do blood testing because of low burden in the area and also worrying about stock out of malaria test kits* [rapid diagnostic tests kits]*.”* (39 year male vector borne disease control staff)
Suggestion to improve timely blood testing among migrants		
Providing needed health message Provider acceptance for malaria testing		*“Everybody will do blood testing timely when the clinics or services available in easily accessible places for example the Dam Gate and needed such kind of health message for timely malaria testing.”*(33 year male charcoal maker) *“However, I would like to suggest the health care providers or volunteer also need to be patient and accept for coming request to do blood testing in every fever cases.”* (33 year male charcoal maker)
Providing peer volunteer with supervision		“*They have* [Migrants] *need to have the information about timely testing within 24* *h of fever. Also providing volunteer in their working site because they will not coming if available sources are far away even they know the information”* (60 year male vector borne disease control staff)
		*“After providing the person who knows well and well trained for malaria in our working places, we suggest community to do timely testing even the fever seems to be malaria or not.”* (51 year male oil digger) *“It is the best to provide Working site leader or other one volunteer from village near work site or in our work site for the purpose of training on blood testing”* (29 year male bamboo cutter)
		*“Peer volunteer is the best and they are always closest one for them. But needs supervision for not becoming quack.”* (56 year male BHS)

Under perceived barriers, 11 themes were identified and broadly grouped into two categories: migrant level (eight themes) and provider level (three themes). The migrant level themes were: inappropriate health care seeking, self-medication, not giving importance to fever, transportation difficulty, not affordable, previous experiences of no timely testing, uninformed about VHV or activities of VHV and lack of symptomatic treatment from a VHV. The provider level themes were: lack of practices for malaria testing, afraid of paper workload and waste of test kits in no burden area.

Under suggested solutions, ‘provider acceptance for malaria testing’ and ‘mobile peer volunteer with supervision’ were the key themes.

## Discussion

This is the first study reporting the extent of malaria testing within 24 h of fever among migrants in Myanmar. There were some key findings.

First, around one in five migrants with fever received a malaria blood test within 24 h. Around two-thirds did not receive a test at all which is concerning. However, testing within 24 h among migrants was significantly higher than general population in Myanmar (12.3%, year 2015) [10]. This was heartening to know because malaria is concentrated among migrants that are one of the high-risk populations. The Myanmar national guidelines recommend detection/treatment and notification of malaria, whether symptomatic or not, within 24 h [4].

Second, factors associated with testing within 24 h were identified. Stable migrants (those belonging to Category I and long intentions to stay) were more likely to be tested within 24 h. This could be explained by relatively better health-seeking among this group of migrants when compared to unstable migrants (data not known). Association with stable migrants (Category I) and appropriate health-seeking from public sector has also been reported among migrants previously [9]. In this study, satisfactory knowledge and appropriate health-seeking also independently contributed to testing within 24 h.

Children less than 5 years are a vulnerable group in high transmission areas; belonging to migrant households further increases their vulnerability to malaria. This group had lower chances of testing within 24 h when compared to other age groups and is of serious concern. The knowledge regarding vulnerable groups was poor among migrant households especially related to this sub-group. Factors for testing within 24 h have not been studied in previous studies either in migrant or in general population. These studies limited themselves to assessing the reasons for health-seeking in public health sector [9, 10].

Third, health-seeking was appropriate in three-fourths of migrants with fever. Knowledge regarding prevention and treatment for malaria was also satisfactory. Despite this, testing within 24 h was seen in one-fifth. The qualitative findings revealed that it was common among migrants to seek appropriate care after 24 h. Many sought care when the fever did not subside. This has also been reported previously among migrants in Myanmar [20]. Few did not get assessed for malaria despite seeking care within 24 h. There were some provider related factors for non-testing which included providing tests preferentially for those who are more ill, fear of stock out and additional paper work. It is possible that in transmission reduction areas, health staff might not have tested some cases of fever. Some migrants also expected symptomatic treatment to be provided by VHV in case malaria was ruled out (this was not done or provided with out of pocket payment).

The presence of VHV is to ensure testing within 24 h. Though there was a question in the migrant malaria survey related to this point (difficulty to visit a VHV), data was missing for majority of migrants with fever. However, there were other findings which pointed towards sub-optimal benefit from VHVs. VHVs were the source of information regarding malaria only in one in five households. Health care was first sought from VHV only in 7% cases while in half it was first sought in township hospital or station hospital or RHC which itself could be far away.

The study has some policy and practice implications. There is a need to improve testing within 24 h. First, there is a need for improving the knowledge related to malaria in general especially the need for testing within 24 h of fever and the option of testing by the nearest available VHV. The existing behaviour change communication strategy for migrants as well as providers should be reviewed for this aspect. Second, the staff at public health facility and VHV should test every case of fever as per guidelines and provide symptomatic treatment as well. The later has been started since 2017 where the VHV has been mandated to provide oral rehydration solution and antipyretics [21]. Finally, the programme may consider peer volunteers trained as VHV among identified mobile population. The same may be linked to the nearest VHV for malaria diagnosis.

### Strengths and limitations

The strengths of the study were as follows (i) the policy and practice implications are from the suggested solutions to address the barriers and the mixed methods design helped us in exploring these, (ii) double data entry and validation of quantitative data minimized data entry errors and (iii) quantitative analysis was weighted for sampling designing making the estimates reliable and robust.

There were some limitations. The following variables were not included in the study as they were not part of the migrant malaria survey 2016: rural–urban status, education status of each individual, availability and distance of VHV for the migrant cluster. Thirteen hard-to-reach townships were excluded and their inclusion would have resulted in lower estimates for testing within 24 h than what has been reported. In the qualitative enquiry, in depth exploration was not done into migrants not visiting the VHV despite knowing that the diagnosis services were available with them and into factors related to not testing among the providers.

## Conclusion

The mixed-methods study identified that blood testing for malaria within 24 h of fever was low among migrants in RAI supported areas of Myanmar. However, this was better than the general population. Satisfactory knowledge, appropriate health care seeking and stable migrant status were associated with undergoing testing within 24 h. Though many sought care appropriately for fever, it was mostly after a few days of fever. This was possibly due to poor utilization of diagnosis services offered by VHV. Barriers and suggested solutions were explored both from the provider as well as migrant perspective. The programme should seriously consider addressing these barriers and implementing the recommendations if Myanmar is to eliminate Malaria by 2030 [4].

## Additional files

**Additional file 1.** Questionnaire of nation-wide migrant malaria survey 2016.

**Additional file 2.** One-to-one interview guide used in the nation-wide migrant malaria survey 2016.

**Additional file 3.** Focus group discussion guide used in the study.

**Additional file 4.** Questions from the nation-wide migrant malaria survey 2016 that contributed to the knowledge score.

## Abbreviations

NMCP: National malaria control programme
RAI: Regional artemisinin-resistance initiative
DMR: Department of medical research
VHV: village health volunteer
BHS: basic health staff
RHC: rural health centre
FGD: focus group discussion
CI: confidence interval
