# Supplementary material for: Low uptake of malaria testing within 24 h of fever despite appropriate health-seeking among migrants in Myanmar: a mixed-methods study
Source: Malar J. 2018 Oct 29;17:396. doi: 10.1186/s12936-018-2546-4 (PMC6206677; doi:10.1186/s12936-018-2546-4)
Supplement: Supplementary file 1 — Additional file 1. Questionnaire of nation-wide migrant malaria survey 2016. [file 12936_2018_2546_MOESM1_ESM.doc]

**S1 Annex**

**Malaria Prevention in Migrant Household Questionnaire (2016)**

**Part (A) Basic Information**

A1 Name of cluster/Ward ------------ A1

A2 Migrant cluster number (1-5) **-----------** A2

A2a Type of Category

1. Category 1
2. Category 2
3. Category 3

### A3 Time required to the nearest heath facility Hour------- Minutes----- A3 hr

min

### A4 Household number (1-50) -- ----- A4

A5 State/ Region --------------- A5

### A6 Township---------------------------- A6

A7 Station/ RHC name------------------ A7

A8 Sub-Center name -------------------- A8

### A9 Name of Head of Household ------------------- A9 _______________

A10 Completeness of interview A10

### 1 = Complete

### 2 = No one at home (No suitable person)

### 3 = Refuse to answer

### 4 = Can’t find the house

### 5 = Other (Specify) __________________

A11 Number of household members ________ A11

A12 Name of respondent _______________ A12 ______________

A13 Household member ID_______ A13

(Line number from household list)

(Choose one person/ Woman is a priority)

| **No.** | **Question** | **Coding Catergories** | **Skips** | **Codes** |
| --- | --- | --- | --- | --- |
| A14 | What is your highest education level?  (Choose one answer) | Illiterate .................................................. 1 Read or write/ Monastic Education........ 2  Primary/ Primary passed  Middle school ..….................................... 3 Middle school passed.............................. 4  High school ………….................................. 5  High school passed.................................. 6  University/ Colledge................................ 7  Graduate/ Post-graduate........................ 8  Don’t know............................................. 98 |  | |___|___| |
| A15 | What is your Race/National? | Myanmar.................................................. 1  Mon ........................................................ 2  Dawei ……................................................ 3  Kayin ..................................................... 4  Kachin ..................................................... 5  Kayin ................................................... 6  Shan ..................................................... 7  Rakhine .................................................... 8  Other (Specify) …………………………………….. 9  ____________________________ |  | |___|  |___| |
| A16 | What is your main occupation? | Rubber plantation.................................... 1  Forest related worker.............................. 2  Farmer …………........................................ 3  Water related worker ............................ 4  Dependent............................................... 5  Other (Specify) .................................. 6  ___________________________ |  | |___|  |___| |
| A17 | What is your working hour? | Daytime (6:00 AM to 6:00 PM) …………... 1  Night time(6:00 PM to 6:00 AM)............ 2 | 🡪A19 | |___| |
| A18 | When do you sleep if you work in the night time? | Night ......... hr ........... min |  | |___|___| hr  |___|___|min |

| A19 | Where were you born? | State/ Region _____________________  Township ______________________  Village cluster/ Village name__________ |  | |____|____|  |____|____|  |____|____| |
| --- | --- | --- | --- | --- |
| A20 | Duration in this township since you arrived here for the first time | ----------- Year ------- Months |  | |___|___| yr  |___|___|mth |
| A21 | Duration in this township since you arrived here for the last time | ----------- Year ------- Months |  | |___|___| yr  |___|___|mth |
| A22 | Are you living here with residence household list or temporary visitor list? | Residence household list ………...................1  Temporary visitor list ............................... 2  Other (Specify) …….................................... 3  __________________________________ |  | |____| |
| A23 | Where did you come from?  (Permanent address) | State/ Region _____________________  Township ______________________  Village cluster/ Village name__________ |  | |____|____|  |____|____|  |____|____| |
| A24 | Why did you move here?  MULTIPLE RESPONSES POSSIBLE  CIRCLE ALL MENTIONED  PROBE ONCE: ANYTHING ELSE? | To work (Already got a job)…………….......... a  To find a job …............................................ b  To be residendce ………............................... c  Visit to relatives………................................. d  Come together with spouse …................. e  Other (Specify) ……..................................... f  __________________________________  Don’t know ............................................. g |  | a |___|  b |___|  c |___|  d |___|  e |___|  f |___|  |___|  g |___| |
| A25 | When do you usually go back home? | Summer .................................................... 1  Rainy season ............................................ 2  Winter ..................................................... 3  Never went back ..................................... 4  Other (Specify).......................................... 5  __________________________________ |  | |___| |
| A26 | On average, how many times do you usually go back home | In 1 year ------------ times  (If never gone back, put “0”) |  | |____|____| |
| A27 | How long do you intend to stay here? | Less than 2 weeks.................................... 1  2weeks – 4 weeks …….............................. 2  1 month – 3 months .............................. 3  3 months – 6 months ............................. 4  6 months – 1 year ……............................... 5  More than 1 year ................................... 6  Not sure ………….................................... 7 |  | |____| |
| A28 | Where do you intend to go next? | Native (Permanent address) ……................ 1  Another workplace, same township ………..2  Another workplace, another township ..... 3  Other (Specify) ……................................... 4  _________________________________  Don’t know ....................................... 5 | B1  B1 | |____| |

I would like to ask some general questions about your house.

| **No.** | **Question** | **Coding Catergories** | **Skips** | **Codes** |  |
| --- | --- | --- | --- | --- | --- |
|  | What is the main material used for roof?  (Circle only ONE answer) | Plastic ...................................................... 1  Thatch/palm/Bamboo............................. 2  Fibre, Aluminium, Iron............................. 3  Zinc, Clay tiles ........................................ 4  Other (Specify) ...................................... 5  ________________________________ |  | |____| |  |
|  | What is the main material used for floor?  (Circle only ONE answer) | Bamboo................................................... 1  Wood ……................................................ 2  Tiles/ Cement ………................................. 3  Earth …................................................... 4  Other (Specify) ……................................. 5  ________________________________ |  | |____| |  |
|  | Do you own this house? | Own house ............................................. 1  Rent ……................................................. 2  Employer allowed to live ……….............. 3  Don’t know ........................................... 4  Other (Specify) ……................................ 5  _________________________________ |  | |____| |  |
|  | During the past 12 months, has anyone sprayed the interior walls of your home against mosquitoes? | Yes ……….................................................. 1  No ………................................................... 2  Don’t know.............................................. 3 | B6  B6 | |____| |  |
|  | Who sprayed the house? | Government worker/ Program …………..... 1  NGO worker/ Program ............................ 2  Private company...................................... 3  Househpld member ............................... 4  Other (Specify) ........................................ 5  Not sure ................................................. 8 |  | |____| | |
|  | What is your main occupation for household income? | Farming ................................................. 1  Gardening.............................................. 2  Agriculture and animal husbandary ….. 3  Merchant .............................................. 4  Daily wager ........................................... 5  Other (Specify) ................................. 6  __________________________________ |  | |____|____| | |
|  | On average, how much do you earn in a month or a year.  (Complete only ONE unit) | Monthly income --------------- kyats  Yearly income -------------- kyats |  | |___| m/yr  |____|____|  |____|____|  |____|____|  |____|____| | |

**Household listing**

Firstly, I would like to ask about your household members. (Ask about each member) (Complete in extra sheet when necessary)

Q5b

1. No net/ no enough net
2. No habit of using net
3. Lazy to use net
4. Go to the forest
5. Visit to others’ house
6. In the hospital
7. Other (Specify) ___________

| Line No. | Give the names of people who usually live in the house and visitors last night (If a baby does not have a name, write babymother's name) | Sex | Age (If less than 1 year of age, write “0” for year) | | Did (NAME) sleep here last night?  (Skip to Q6 if did not use) | Did (NAME) use mosquito net last night?  (Skip to Q6 if did not use) | Reason for not sleeping with mosquito net. | Is (NAME) currently pregnant? |
| --- | --- | --- | --- | --- | --- | --- | --- | --- |
|  |  |  |  | |  | Q5a | Q5b |  |
| Line No. | Name | M F | Year (Completed) | Month | Yes No | Yes No | Add codes* | Yes No Don’t know |
| 01 |  | 1 2 |  |  | 1 2 | 1 2 |  | 1 2 8 |
| 02 |  | 1 2 |  |  | 1 2 | 1 2 |  | 1 2 8 |
| 03 |  | 1 2 |  |  | 1 2 | 1 2 |  | 1 2 8 |
| 04 |  | 1 2 |  |  | 1 2 | 1 2 |  | 1 2 8 |
| 05 |  | 1 2 |  |  | 1 2 | 1 2 |  | 1 2 8 |
| 06 |  | 1 2 |  |  | 1 2 | 1 2 |  | 1 2 8 |
| 07 |  | 1 2 |  |  | 1 2 | 1 2 |  | 1 2 8 |
| 08 |  | 1 2 |  |  | 1 2 | 1 2 |  | 1 2 8 |
| 09 |  | 1 2 |  |  | 1 2 | 1 2 |  | 1 2 8 |
| 10 |  | 1 2 |  |  | 1 2 | 1 2 |  | 1 2 8 |
| 11 |  | 1 2 |  |  | 1 2 | 1 2 |  | 1 2 8 |
| 12 |  | 1 2 |  |  | 1 2 | 1 2 |  | 1 2 8 |

Knowledge on Malaria

| **No.** | **Question** | **Coding Catergories** | **Skips** | **Codes** |
| --- | --- | --- | --- | --- |
|  | Have you ever heard of malaria?  IF ANSWER IS "NO", REPEAT ASKING QUESTION IN LOCAL COLLOQUIAL WORD FOR MALARIA | Yes ....................................................... 1  No ........................................................ 2 | 🡺**Q24** | |___| |
|  | What are the causes of malaria?  MULTIPLE RESPONSES POSSIBLE  CIRCLE ALL MENTIONED  PROBE ONCE: ANYTHING ELSE? | Mosquito bite …................................... a  Drinking dirty water ……...................... b  Eating bananas .................................... c  Bad food ……………................................ d  Working in the forest/ farm… ………..... e  Witch/ Devil spirits............................... f  Poor hygeine ....................................... g  Bad air ................................................. h  Other (Specify)..................................... y  _______________________________  _______________________________  Don’t know ......................................... z |  | a |___|  b |___|  c |___|  d |___|  e |___|  f |___|  g |___|  h |___|  y |___|  |__|__|  |__|__|  z |___| |
|  | How can malaria be transmitted from person to person?  MULTIPLE RESPONSES POSSIBLE  CIRCLE ALL MENTIONED  PROBE ONCE: ANYTHING ELSE? | Mosquito bite ..................................... a  Drinking water .................................... b  Eating together.................................... c  Same blood group ............................... d  Mother to child ................................... e  Staying together .................................. f  Sexual intercourse ............................... g  Sharing same bed ................................. h  Other (Specify) ...................................... y  ________________________________  ________________________________  Don’t know............................................ z |  | a |___|  b |___|  c |___|  d |___|  e |___|  f |___|  g |___|  h |___|  y |___|  |__|__|  |__|__|  z |___| |
|  | Do you think malaria is a fatal disease? | Yes .......................................................... 1  No .......................................................... 2  Don’t know ............................................ 3 |  | |___| |
|  | What are the signs or symptoms of malaria?  MULTIPLE RESPONSES POSSIBLE  CIRCLE ALL MENTIONED  PROBE ONCE: ANYTHING ELSE? | Fever ...................................................... a  Chills and rigors ...................................... b  Excessive sweating ................................. c  Whole body ache ................................... d  Anaemia ................................................. e  Splenomegaly ......................................... f  Loss of appetite/ Vomiting...................... g  Reduce urine output ............................... h  High colored urine/ Black water fever …. i  Jaundice .................................................. j  None of above …..................................... k  Other (Specify) ....................................... y  ________________________________  ________________________________  Don’t know ............................................. z |  | a |___|  b |___|  c |___|  d |___|  e |___|  f |___|  g |___|  h |___|  i |___|  j |___|  k |___|  y |___|  |__|__|  |__|__|  z |___| |
|  | What is the vulnerable age group for malaria?  MULTIPLE RESPONSES POSSIBLE  CIRCLE ALL MENTIONED | Less than 5 yrs old .............................. a  Pregnant mother ................................ b Farmers .............................................. c  Forest related workers ………………......d  Other (Specify) ................................... y  ________________________________  Don’t know.......................................... z |  | a |___|  b |___|  c |___|  d |___|  y |___|  |__|__|  z |___| |
|  | Can malaria be prevented? | Yes 1  No 2  Don’t know 8 | 🡺**Q15** | |___| |
|  | How can malaria be prevented?  MULTIPLE RESPONSES POSSIBLE  CIRCLE ALL MENTIONED  PROBE ONCE: ANYTHING ELSE?  **Note: If choose sleeping under mosquito net, ask the type of net to know the knowledge about ITN** | Sleeping under mosquito net ..................a  Using ITN/LLIN .......................................b  Using modern medicine .........................c  Mosquito coil/spray/repellent ………..…..d  Burning leaves/tumeric...........................e  Drinking boiled water .............................f  Wearing long sleeve clothes ...................g  Stay out of forest ....................................h  Other (Specify) .......................................y  __________________________________  __________________________________  Don’t know........................................... z |  | a |___|  b |___|  c |___|  d |___|  e |___|  f |___|  g |___|  h |___|  y |___|  |__|__|  |__|__|  z |___| |
|  | Can malaria be treated? | Yes 1  No 2  Don’t know 8 | 🡺**Q17** | |___| |
|  | How can malaria be treated?  MULTIPLE RESPONSES POSSIBLE  CIRCLE ALL MENTIONED  PROBE ONCE: ANYTHING ELSE? | Traditional medicine ............................... a  Western medicine................................... b  Both traditional and western medicine …c  Other (Specify) ........................................y  __________________________________  Don’t know.............................................. z |  | a |___|  b |___|  c |___|  y |___|  |__|__|  z |___| |
|  | Have you ever heard or seen any messages or information about malaria? | Yes 1  No 2 | 🡺Q24 | |___| |
|  | How long ago did you see or hear these messages for the last time? | Previous ------ month  ------ year |  | |___|___|  |___|___| |
|  | What messages or information related to malaria did you see or hear?  MULTIPLE RESPONSES POSSIBLE  CIRCLE ALL MENTIONED  PROBE ONCE: ANYTHING ELSE? | Sleeping under a mosquito net is  Important …………………………………………..a  Sleep under an insecticide-treated  net (ITN) …………………………………….…….. b  Carry and sleep under a mosquito  net when traveling……………………………..c  Carry and sleep under a mosquito  net when visiting the forest ……..………..d  Seek treatment for malaria from a  VMW or health facility ……………………….. e  Seek treatment for malaria  promptly/within 24 hours ………..……….. f  Complete antimalarial treatment…………..g  Get a blood test before taking  antimalarial drugs………………………………..h  Malaria is dangerous…………………….………..i  Malaria can kill………………………………..……..j  Mosquitoes spread malaria……….…………..k  Other (Specify)………………………………..……..y  ________________________________  _________________________________  Don't Remember ……………………..………….. z |  | a |___|  b |___|  c |___|  d |___|  e |___|  f |___|  g |___|  h |___|  i |___|  j |___|  k |___|  y |___|  |__|__|  |__|__|  z |___| |
|  | Where or from whom did you see or hear these messages/information about malaria?  MULTIPLE RESPONSES POSSIBLE  CIRCLE ALL MENTIONED  PROBE ONCE: ANYWHERE ELSE? | VMW/VHV ………………………………………….. a  Health facility staff ….………………………….. b  Private health provider ……………………….. c  Pharmacy …………………………..……………….. d  Teachers ……………………………….…………….. e  Religious leaders/Monks …………………….. f  Family members ..……………………………….. g  Friends/neighbours …………………………….. h  TV ……………………………………………………….. i  Radio ………………………………………………….. j  Video ……………………………………..………….. k  Posters ……………………………………………….. l  Leaflets/Brochures …………………….………..m  Billboards ………………………………………….. n  Other (Specify) …………………………………… y  _______________________________  Don't Remember………………………….. z |  | a |___|  b |___|  c |___|  d |___|  e |___|  f |___|  g |___|  h |___|  i |___|  j |___|  k |___|  l |___|  m|___|  n |___|  y |___|  |__|__|  z |___| |
|  | Have you ever heard or seen any information about prevention of anti-malarial drugs resistance?  (Explain about drug resistance when necessary) | Yes 1  No 2 | 🡺**Q24** | |___| |
|  | What did you hear about the prevention of anti-malarial drug resistance?  MULTIPLE RESPONSES POSSIBLE  CIRCLE ALL MENTIONED  PROBE ONCE: ANYWHERE ELSE? | Sleep under an insecticide treated net (ITN) ..................................................... a  Prevent from mosquito bite while working in the forest ........................................... b  Get malaria treated by a VHV or a health facility ……………………………………………….. c  Seek treatment for malaria  promptly/within 24 hours ...................d  Complete anti-malarial treatment ………. e  Avoid taking medicine containing Artemisinin alone .................................. f  Other (Specigy) ...................................... y  ________________________________  Don’t remember.................................... z |  | a |___|  b |___|  c |___|  d |___|  e |___|  f |___|  y |___|  |__|__|  z |___| |
|  | Where did you get the information about the prevention of anti-malarial drug resistance?  MULTIPLE RESPONSES POSSIBLE  CIRCLE ALL MENTIONED  PROBE ONCE: ANYWHERE ELSE? | VMW/VHV ………………………………………….. a  Health facility staff ….………………………….. b  Private health provider ……………………….. c  Pharmacy …………………………..……………….. d  Teachers ……………………………….…………….. e  Religious leaders/Monks …………………….. f  Family members ..……………………………….. g  Friends/neighbours …………………………….. h  TV ……………………………………………………….. i  Radio ………………………………………………….. j  Video ……………………………………..………….. k  Posters ……………………………………………….. l  Leaflets/Brochures …………………….………..m  Billboards ………………………………………….. n  Other (Specify) …………………………………… y  _______________________________  Don't Remember………………………..………..z |  | a |___|  b |___|  c |___|  d |___|  e |___|  f |___|  g |___|  h |___|  i |___|  j |___|  k |___|  l |___|  m|___|  n |___|  y |___|  |__|__|  z |___| |
|  | Have you ever heard of insecticide treated net? | Yes 1  No ...................................................... 2 | 🡺**Q29** | |___| |
|  | What are the benefits of a mosquito net treated with insecticide compared to an untreated net?  MULTIPLE RESPONSES POSSIBLE  CIRCLE ALL MENTIONED  PROBE ONCE: ANYWHERE ELSE? | Prevents mosquito bites a  Prevents squito transmitted diseases b  Prevents malaria c  Kills other insects d  Kills mosquito e  Better sleep f  Other (Specify) y  ______________________________  Don't know z |  | a |___|  b |___|  c |___|  d |___|  e |___|  f |___|  y |___|  |__|__|  z |___| |
|  | Have you ever heard or seen some information about making insecticide treated nets long lasting? | Yes ...................................... 1  No ......................................... 2 | 🡺**Q29** | |___| |
|  | What did you hear about making insecticide treated nets long lasting?  MULTIPLE RESPONSES POSSIBLE  CIRCLE ALL MENTIONED  PROBE ONCE: ANYWHERE ELSE? | Can wash up to 20 times ....................... a  Soak with insecticide again after ........... b  ______ months  (Write “0” if less than 1 month)  Dry in shade/Not in the sun ……………...... c  Wash gently/Don’t wash with stick......... d  Don’t wash with soap............................. e  Other (Specify)........................................ y  ______________________________  Don’t remember..................................... z |  | a |___|  b |___|  |___|  c |___|  d |___|  e |___|  y |___|  |__|__|  z |___| |
|  | Where did you hear about making insecticide treated nets long lasting?  MULTIPLE RESPONSES POSSIBLE  CIRCLE ALL MENTIONED  PROBE ONCE: ANYWHERE ELSE? | VMW/VHV ………………………………………….. a  Health facility staff ….………………………….. b  Private health provider ……………………….. c  Pharmacy …………………………..……………….. d  Teachers ……………………………….…………….. e  Religious leaders/Monks …………………….. f  Family members ..……………………………….. g  Friends/neighbours …………………………….. h  TV ……………………………………………………….. i  Radio ………………………………………………….. j  Video ……………………………………..………….. k  Posters ……………………………………………….. l  Leaflets/Brochures …………………….………..m  Billboards ………………………………………….. n  Other (Specify) …………………………………… y  _______________________________  Don't Remember………………………..………..z |  | a |___|  b |___|  c |___|  d |___|  e |___|  f |___|  g |___|  h |___|  i |___|  j |___|  k |___|  l |___|  m|___|  n |___|  y |___|  |__|__|  z |___| |

**Part (C) Human behavior related to malaria**

**Practice and behaviours related to fever within 3 months**

| **No.** | **Questions** | **Coding Catergories** | **Skips** | **Codes** |
| --- | --- | --- | --- | --- |
|  | Has anyone in the household been ill with a fever during the last 3 months? | Yes 1  No ................................................... 2  Don’t know ......................................... 8 | 🡺**Q39** | |___| |
|  | How many people in the household have been ill with fever during the last two weeks? | Number of febrile persons ________ |  | |___|___| |

**Use extra sheet for fever if more than 2 febrile persons present**

| **No.** | **Questions** | **Fever 1** | **Fever 2** |
| --- | --- | --- | --- |
|  | Who was ill with fever in the last 3 months? | Name ___________  Line No. | Name ___________  Line No. |
|  | In which month did (NAME) ill with fever? | 1. 1 month ago |___| 2. 2 months ago |___| 3. 3 months ago |___| | 1. 1 month ago |___| 2. 2 months ago |___| 3. 3 months ago |___| |
| Q32a | Which kind of illness did you think ?  MULTIPLE RESPONSES POSSIBLE  CIRCLE ALL MENTIONED | 1. Common cold |___| 2. Abscess/Ulcer |___| 3. Malaria |___| 4. Other (Specify) |___|   ___________________ | 1. Common cold |___| 2. Abscess/Ulcer |___| 3. Malaria |___| 4. Other (Specify) |___|   ___________________ |
|  | Is it easy for you to visit to a malaria staff (or) VHV?  Can you reach them within 24 hours? | 1. Yes. Can reach within 24 hours 2. Yes. But can’t reach within 24 hours in rainy season 3. No. But can reach within 24 hours 4. Not at all 5. Don’t know | 1. Yes. Can reach within 24 hours 2. Yes. But can’t reach within 24 hours in rainy season 3. No. But can reach within 24 hours 4. Not at all 5. Don’t know |
|  | Why is it difficult to visit to visit to a VHV or a health facility? | 1. Come from more than 3 miles distance 2. Don’t think feer is important 3. No money to seek treatment 4. Busy 5. Difficulty in transportation 6. Don’t know there is malaria staff or VHV 7. Other (Specify) ____________ | 1. Come from more than 3 miles distance 2. Don’t think feer is important 3. No money to seek treatment 4. Busy 5. Difficulty in transportation 6. Don’t know there is malaria staff or VHV 7. Other (Specify) ____________ |
|  | Where did you seek the **first** treatment? | No medication ............................. 0  Self-medication ........................... 1  Traditional healer......................... 2  Tranined VHV ……………………………. 3  RHC/ Sub-center ........................ 4  Township/ Station hospital.......... 5  Private clinic ............................... 6  Private clinic of midwife/ HA …….. 7  AMW ............................................ 8  Other (Specify) ............................. 9  _____________  Don’t know .................................. 98 | No medication ............................. 0  Self-medication ........................... 1  Traditional healer......................... 2  Tranined VHV ……………………………. 3  RHC/ Sub-center ........................ 4  Township/ Station hospital.......... 5  Private clinic ............................... 6  Private clinic of midwife/ HA …….. 7  AMW ............................................ 8  Other (Specify) ............................. 9  _____________  Don’t know .................................. 98 |
|  | Where did you seek the **second** treatment if not relieved by the first time? | No medication ............................. 0  Self-medication ........................... 1  Traditional healer......................... 2  Tranined VHV ……………………………. 3  RHC/ Sub-center ........................ 4  Township/ Station hospital.......... 5  Private clinic ............................... 6  Private clinic of midwife/ HA …….. 7  AMW ............................................ 8  Other (Specify) ............................. 9  _____________  Don’t know .................................. 98 | No medication ............................. 0  Self-medication ........................... 1  Traditional healer......................... 2  Tranined VHV ……………………………. 3  RHC/ Sub-center ........................ 4  Township/ Station hospital.......... 5  Private clinic ............................... 6  Private clinic of midwife/ HA …….. 7  AMW ............................................ 8  Other (Specify) ............................. 9  _____________  Don’t know .................................. 98 |
|  | Where did you seek the **third** treatment if not relieved by the second time? | No medication ............................. 0  Self-medication ........................... 1  Traditional healer......................... 2  Tranined VHV ……………………………. 3  RHC/ Sub-center ........................ 4  Township/ Station hospital.......... 5  Private clinic ............................... 6  Private clinic of midwife/ HA …….. 7  AMW ............................................ 8  Other (Specify) ............................. 9  _____________  Don’t know .................................. 98 | No medication ............................. 0  Self-medication ........................... 1  Traditional healer......................... 2  Tranined VHV ……………………………. 3  RHC/ Sub-center ........................ 4  Township/ Station hospital.......... 5  Private clinic ............................... 6  Private clinic of midwife/ HA …….. 7  AMW ............................................ 8  Other (Specify) ............................. 9  _____________  Don’t know .................................. 98 |
|  | Did you have blood testing for malaria? | Yes .......................................... 1  No............................................ 2  Don’t know/Not sure...............8  (If Don’t know/Not sure, go to **Q39** ) | Yes .......................................... 1  No............................................ 2  Don’t know/Not sure...............8  (If Don’t know/Not sure, go to **Q39** ) |
|  | On which day of fever did yu do blood testing for malaria? | Within 24 hours............................ 1  Within 24-48 hours ……................ 2  After 48 hours (specify)…............. 3 (Days from 1^st^ day of fever___ Days)  Not sure....................................... 8 | Within 24 hours............................ 1  Within 24-48 hours ……................ 2  After 48 hours (specify)…............. 3 (Days from 1^st^ day of fever___ Days)  Not sure....................................... 8 |
|  | What is the results? | Positive ........................................ 1  Negative....................................... 2  Don’t know/Not sure................... 8 | Positive ........................................ 1  Negative....................................... 2  Don’t know/Not sure................... 8 |

**Household Nets**

| **No.** | **Questions** | **Coding Categories** | **Skips** | **Codes** |
| --- | --- | --- | --- | --- |
|  | Does your household have any mosquito nets (bed nets or hammock nets) that can be used while sleeping? | Yes ............................................ 1  No ............................................. 2  Don’t know ............................... 8 | 🡺**Q55** | |___| |
|  | How many mosquito nets does your household have?  ALSO INCLUDE ANY THAT ARE NOT CURRENTLY IN USE | Tota number of mosquito nets:  (exclude Hammock nets) ______ |  | |  |  | | --- | --- | |
|  |  | Number of hammock nets: ______ |  | \|  \|  \| \| --- \| --- \| |

**Observe the nets and record the number of nets and check the holes**

**Use extra sheet for nets if number of net exceeds 3**

| **No.** | **Questions** | **Net 1** | **Net 2** | **Net 3** |
| --- | --- | --- | --- | --- |
|  | Did you observed the mosquito net? | Observed 1  Not observed 2 | Observed 1  Not observed 2 | Observed 1  Not observed 2 |
|  | How long ago did your household obtain this net? | < 6 months 1  6 mo to <1 year 2  1 yr to < 2 yr 3  2 yr to < 3 yr 4  3 yr to < 5 yr 5  ≥ 5 years 6  Don't know 8 | < 6 months 1  6 mo to <1 year 2  1 yr to < 2 yr 3  2 yr to < 3 yr 4  3 yr to < 5 yr 5  ≥ 5 years 6  Don't know 8 | < 6 months 1  6 mo to <1 year 2  1 yr to < 2 yr 3  2 yr to < 3 yr 4  3 yr to < 5 yr 5  ≥ 5 years 6  Don't know 8 |
|  | Type of net  (**If answered LLIN, continue asking Q44. If not, skip to Q45**) | Cotton .................... 1  Nylon...................... 2  CLace ................... 3  CYC cotton ............. 4  Military net ………….. 5  LLIN..............6 Q44  Other (specify) ………7  ______  Don’t know ........... 8 | Cotton .................... 1  Nylon...................... 2  CLace ................... 3  CYC cotton ............. 4  Military net ………….. 5  LLIN..............6 Q44  Other (specify) ………7  ______  Don’t know ........... 8 | Cotton .................... 1  Nylon...................... 2  CLace ................... 3  CYC cotton ............. 4  Military net ………….. 5  LLIN..............6 Q44  Other (specify) ………7  ______  Don’t know ........... 8 |
|  | If LLIN, where did your household obtain this net? | Gift (family/friend)….1  Government…………..2  NGO………………………3  Shop/Market………….4  Itinerant seller ……….5  Other…………………….6  ________________  Don't know…………….8 | Gift (family/friend)….1  Government…………..2  NGO………………………3  Shop/Market………….4  Itinerant seller ……….5  Other…………………….6  ________________  Don't know…………….8 | Gift (family/friend)….1  Government…………..2  NGO………………………3  Shop/Market………….4  Itinerant seller ……….5  Other…………………….6  ________________  Don't know…………….8 |
|  | Size of net | Single size................. 1  Single + half ........... 2  Two persons .......... 3  Family size................ 4 | Single size................. 1  Single + half ........... 2  Two persons .......... 3  Family size................ 4 | Single size................. 1  Single + half ........... 2  Two persons .......... 3  Family size................ 4 |
|  | Condition of net | Good (no holes)........1  Reparied (no holes)...2  Small holes ..............3  Bad (Large holes)......4 | Good (no holes)........1  Reparied (no holes)...2  Small holes ..............3  Bad (Large holes)......4 | Good (no holes)........1  Reparied (no holes)...2  Small holes ..............3  Bad (Large holes)......4 |
|  | Has any of your net ever been soaked or dipped in a liquid to kill/repel mosquitoes? | Yes 1  No 2  Not sure 8  IF NO, SKIP TO **Q49** | Yes 1  No 2  Not sure 8  IF NO, SKIP TO **Q49** | Yes 1  No 2  Not sure 8  IF NO, SKIP TO **Q49** |
|  | How long since the net was last soaked or dipped in a liquid to kill/repel mosquitoes? | < 6 months………………1  6 mo to <1 year……….2  1 yr to < 2 yr……………3  2 yr to < 3 yr…………..4  3 yr to < 5 yr…………..5  ≥ 5 years………………..6  Don't know…………….8 | < 6 months………………1  6 mo to <1 year……….2  1 yr to < 2 yr……………3  2 yr to < 3 yr…………..4  3 yr to < 5 yr…………..5  ≥ 5 years………………..6  Don't know…………….8 | < 6 months………………1  6 mo to <1 year……….2  1 yr to < 2 yr……………3  2 yr to < 3 yr…………..4  3 yr to < 5 yr…………..5  ≥ 5 years………………..6  Don't know…………….8 |
|  | How frequently has this mosquito net been washed since you received it? | Weekly ………………….1  Every 2-3 weeks..…..2  Monthly………………….3  Every 2-3 months…..4  Twice per year……….5  Once per year………..6  < Once a year…………7  Never…………………….0  Not sure…………………8  If Never, Go to **Q45** | Weekly ………………….1  Every 2-3 weeks..…..2  Monthly………………….3  Every 2-3 months…..4  Twice per year……….5  Once per year………..6  < Once a year…………7  Never…………………….0  Not sure…………………8  If Never, Go to **Q45** | Weekly ………………….1  Every 2-3 weeks..…..2  Monthly………………….3  Every 2-3 months…..4  Twice per year……….5  Once per year………..6  < Once a year…………7  Never…………………….0  Not sure…………………8  If Never, Go to **Q45** |
|  | What soap do you use to wash LLIN? | 1. soap 2. Washing powder/liquid/gel | 1. soap 2. Washing powder/liquid/gel | 1. soap 2. Washing powder/liquid/gel |
|  | How do you wash LLIN? | 1. With hands 2. With sticks 3. Press with legs 4. Other(Specify)   ________________ | 1. With hands 2. With sticks 3. Press with legs 4. Other(Specify)   ________________ | 1. With hands 2. With sticks 3. Press with legs 4. Other(Specify)   ________________ |
|  | Where do you usually hang this mosquito net after washing? (Decide in the sun or away from direct sun light) | Away from direct sun light......................... 1  In the sun ............... 2  Not sure................... 8 | Away from direct sun light......................... 1  In the sun ............... 2  Not sure................... 8 | Away from direct sun light......................... 1  In the sun ............... 2  Not sure................... 8 |
|  | Did anyone sleep under this mosquito net **last night**? | Yes………………………….1  No…………………………..2  Not sure………………….8  IF NO/Unsure, SKIP TO **Q55** | Yes………………………….1  No…………………………..2  Not sure………………….8  IF NO/Unsure, SKIP TO **Q55** | Yes………………………….1  No…………………………..2  Not sure………………….8  IF NO/Unsure, SKIP TO **Q55** |
|  | Who slept under this mosquito net last night?  ASK FOR THE NAME AND AGE OF EACH PERSON WHO SLEPT UNDER THE NET, THEN CHECK SECTION 1 FOR THE LIST OF HOUSEHOLD MEMBERS AND VISITORS AND WRITE THEIR LINE CODE (Q1) | 1) Name__________  Line code:  2) Name__________  Line code:  3) Name__________  Line code:  4) Name__________  Line code:  5) Name__________  Line code:  6) Name__________  Line code: | 1) Name__________  Line code:  2) Name__________  Line code:  3) Name__________  Line code:  4) Name__________  Line code:  5) Name__________  Line code:  6) Name__________  Line code: | 1) Name__________  Line code:  2) Name__________  Line code:  3) Name__________  Line code:  4) Name__________  Line code:  5) Name__________  Line code:  6) Name__________  Line code: |

| **No.** | **Questions** | **Coding Categories** | **Skips** | **Codes** |
| --- | --- | --- | --- | --- |
|  | If you want to soak your net with insecticide, what would you do?  CIRCLE ONLY **ONE** ANSWER | Wait for the program staffs……………..1  VHW/VHV ………….............................. 2  Other (Specify) ................................. 3 |  | |___| |
|  | What are the benefits of ITN/LLIN over ordinary nets?  MULTIPLE RESPONSES POSSIBLE  CIRCLE ALL MENTIONED  PROBE ONCE: ANYTHING ELSE? | No benefit ........................................ a  Prevent/kill mosquitos..................... b Kill begs/lice...................................... c  Prevent malaria................................ d  aoG;vGefwkwfauG;umuG,f ............... e  Other (Specify) …............................... y  ______________________________  Don’t know........................................ z |  | a |___|  b |___|  c |___|  d |___|  e |___|  y |___|  |___|___|  z |___| |
|  | **Have you ever heard of soaking net or giving nets in this area?** | **Yes.......................................... 1**  **No ......................................... 2** | 🡺**Q60** | |___| |
|  | **Have you ever received net or got your net soaked with insecticides?** | **Net being soaked ………………..**........... 1  **Received net…………..**........................ 2  **Both…….………………………..………………..** 3  **Nothing** ........................................... 4  **Not sure** .......................................... 5 | 🡺**Q60**  🡺**Q60**  🡺**Q60** | |___| |
|  | **Why didn’t you receive anything?** | **In the work**......................................... 1  **On the trip**.......................................... 2  **Not included in the list**……………………..3  **Other (Specify)** .................................. 4  **______________________________** |  | |___| |

**Forest going behaviour**

| **No.** | **Questions** | **Coding Categories** | **Skips** | **Codes** |
| --- | --- | --- | --- | --- |
|  | Does anyone in your household go to the forest and sleep there overnight in the past 3 months?  (for example – farming, gold mine, wood cutting, charcoal making, rubber plantation) | Yes....................................... 1  No........................................ 2  Don’t know............................ 8 | 🡺**Finished** | |___| |
|  | What the forest goer(s) did in the forest?  (Single response- choose main answer) | Farming ................................... 1  Gold/Jade mine .......................... 2  Wood cutting ............................. 3  Charcoal making.......................... 4  Rubber plantation ...................... 5  Seller .......................................... 6  Other(specify) ............................ 7  ___________________________  ___________________________  Don't know ............................. 98 |  | 1|___|  2|___|  3|___|  4|___|  5|___|  6|___|  7|___|  |___|___|  |___|___|  98|___| |
|  | How many people in your household sometimes go to the forest and sleep there overnight in the **past 3 months**? | Number of people ____________ |  | |___|___| |

**Use extra sheet if number of person exceeds 3**

| **No.** | **Questions** | **Person 1** | **Person 2** | **Person 3** |
| --- | --- | --- | --- | --- |
|  | Who in your household sometimes go to the forest and sleeps there overnight in the past 3 months? | Name ___________  Line code: | Name ____________  Line code: | Name ____________  Line code: |
|  | Did (NAME) usually take a mosquito net with them when they went to the forest? | Yes………………………1  No ……………………..2  Not sure …………….8  **IF NO, SKIP TO Q67**  **IF Unusre, SKIP TO Q68** | Yes………………………1  No ……………………..2  Not sure …………….8  **IF NO, SKIP TO Q67**  **IF Unusre, SKIP TO Q68** | Yes………………………1  No ……………………..2  Not sure …………….8  **IF NO, SKIP TO Q67**  **IF Unusre, SKIP TO Q68** |
|  | What type of net did (NAME) use in the forest last time? | Plain mosquito net 1  ITN 2  LLIN 3  Not sure 8 | Plain mosquito net 1  ITN 2  LLIN 3  Not sure 8 | Plain mosquito net 1  ITN 2  LLIN 3  Not sure 8 |
|  | Did (NAME) use a mosquito net the last time they went to the forest? | Yes 1  No 2  Not sure 8  **IF YES, SKIP TO Q68** | Yes 1  No 2  Not sure 8  **IF YES, SKIP TO Q68** | Yes 1  No 2  Not sure 8  **IF YES, SKIP TO Q68** |
|  | If (NAME) did not take a net with them to the forest last time or did not use it, what was the reason why?  MULTIPLE RESPONSES,  CIRCLE ALL MENTIONED  PROBE: ANYTHING ELSE? | Didn't want to use ..a  Forgot to take ………b  Not enough nets in  house c  Nowhere to hang in  forest d  No money to buy e  Other(Specify) ……y  ________________  Don't know z | Didn't want to use ..a  Forgot to take ………b  Not enough nets in  house c  Nowhere to hang in  forest d  No money to buy e  Other(Specify) ……y  ________________  Don't know z | Didn't want to use ..a  Forgot to take ………b  Not enough nets in  house c  Nowhere to hang in  forest d  No money to buy e  Other(Specify) ……y  ________________  Don't know z |
|  | Did (NAME) take any other action to avoid getting malaria?  MULTIPLE RESPONSES,  CIRCLE ALL MENTIONED  PROBE: ANYTHING ELSE? | Mosquito coil 1  Repellent 2  Boiled water 3  Burned leaves 4  Took medication 5  Wore long clothes 6  No action taken 7  Other (Specify)…….8  ________________  Don't know 98 | Mosquito coil 1  Repellent 2  Boiled water 3  Burned leaves 4  Took medication 5  Wore long clothes 6  No action taken 7  Other (Specify)…….8  ________________  Don't know 98 | Mosquito coil 1  Repellent 2  Boiled water 3  Burned leaves 4  Took medication 5  Wore long clothes 6  No action taken 7  Other (Specify)…….8  ________________  Don't know 98 |
|  | Did (NAME) get sick while away from home? | Yes 1  No 2  Don't know 8  **IF NO, Finished** | Yes 1  No 2  Don't know 8  **IF NO, Finished** | Yes 1  No 2  Don't know 8  **IF NO, Finished** |
|  | Where did you seek for treatment when you have fever in the forest? | Self treated……………...1  Traditional healer ….. 2  VHW ……………….….....3  RHC ………………..……...4  Township/Station Hospital ………….…..... 5  GP clinic ……………..…..6  Private clinic………..…..7  AMW……..............…...8  Other (specify)……......9  _______________  Don’t know ……………98 | Self treated……………...1  Traditional healer ….. 2  VHW ……………….….....3  RHC ………………..……...4  Township/Station Hospital ………….…..... 5  GP clinic ……………..…..6  Private clinic………..…..7  AMW……..............…...8  Other (specify)……......9  _______________  Don’t know ……………98 | Self treated……………...1  Traditional healer ….. 2  VHW ……………….….....3  RHC ………………..……...4  Township/Station Hospital ………….…..... 5  GP clinic ……………..…..6  Private clinic………..…..7  AMW……..............…...8  Other (specify)……......9  _______________  Don’t know ……………98 |
|  | Did (Name) take blood test for malaria? | Yes……………….1  No………………..2  Don’t know/  not sure ………….....8  (if No/don’t know/not sure, questions go to end) | Yes……………….1  No………………..2  Don’t know/  not sure ………….....8  (if No/don’t know/not sure, questions go to end) | Yes……………….1  No………………..2  Don’t know/  not sure ………….....8  (if No/don’t know/not sure, questions go to end) |
|  | On which day of fever did yu do blood testing for malaria? | Within 24hour .......................... 1  Within 24-48 hours ……...................... 2  After 48 hours (specify)…............. 3 (Days from 1^st^ day of fever___ Days)  Not sure ...............8 | Within 24hour .......................... 1  Within 24-48 hours ……...................... 2  After 48 hours (specify)…............. 3 (Days from 1^st^ day of fever___ Days)  Not sure ............... 8 | Within 24hour .......................... 1  Within 24-48 hours ……....................2  After 48 hours (specify)…..........3 (Days from 1^st^ day of fever___ Days)  Not sure ...........8 |
|  | What is the results? | Positive ................1  Negative...............2  Don’t know/  Not sure................... 8 | Positive ................1  Negative...............2  Don’t know/  Not sure................... 8 | Positive ................1  Negative...............2  Don’t know/  Not sure................... 8 |

**Interviewer’s signature ......................... Supervisor’s signature.......................**

**Name .................................. Name ...................................**

**Interview Date ......................... Review Date .......................**

**Check start to end!**
