# Supplementary material for: Low uptake of malaria testing within 24 h of fever despite appropriate health-seeking among migrants in Myanmar: a mixed-methods study
Source: Malar J. 2018 Oct 29;17:396. doi: 10.1186/s12936-018-2546-4 (PMC6206677; doi:10.1186/s12936-018-2546-4)
Supplement: Supplementary file 2 — Additional file 2. One-to-one interview guide used in the nation-wide migrant malaria survey 2016. [file 12936_2018_2546_MOESM2_ESM.docx]

Key Informant Interview/In-Depth Interview for provider side: Interview Guide

Name of the participant:

Designation:

Date of Interview:

Interview start / end time:

Name of the Interviewer:

After a brief introduction to the participant regarding the purpose of the interview, the principal investigator will take informed written consent for the interview. Written informed consent will also be requested for audio recording

Theme 1 Current activities of malaria diagnosis for migrant population

Could you please explain me current activities of malaria diagnosis for migrant population?

Which organizations are involved? Who are assigned for malaria diagnosis? How they are trained? How do they perform malaria diagnosis?

Could you please explain me current activities of malaria diagnosis for migrant population by village malaria workers?

Theme 2 Malaria diagnosis and treatment has been initiated by village malaria workers. What do you think are the positive points and negative points about Malaria diagnosis and treatment services provided by village malaria workers?

Theme 3 barriers and challenges in malaria testing for migrant population

• Accessibility, affordability, acceptance by client

• Issues on manpower, budget allocation, equipment supply

• Capacity build of resources

• Are there any reasons regarding non / late initiation of uptake of malaria testing within 24 hours onset of fever among migrant population? [Probe: programmatic, patient-level]

• What are the operational issues involved in this: both at provider level and patient level?

Theme 4 How can we further improve the early uptake of malaria testing among migrant population?

Additional remarks, if any?

Principal investigator will complete the interview by acknowledging the time spared by the participant from his/her busy schedule. He will also give a summary of the notes taken and confirm the same from the participant.
