# Supplementary material for: Low uptake of malaria testing within 24 h of fever despite appropriate health-seeking among migrants in Myanmar: a mixed-methods study
Source: Malar J. 2018 Oct 29;17:396. doi: 10.1186/s12936-018-2546-4 (PMC6206677; doi:10.1186/s12936-018-2546-4)
Supplement: Supplementary file 3 — Additional file 3. Focus group discussion guide used in the study. [file 12936_2018_2546_MOESM3_ESM.docx]

Focus Group Discussion for client side: Interview Guide

Date of Interview:

Interview start / end time:

Name of the Interviewer:

After a brief introduction to the participant regarding the purpose of the interview, the principal investigator will take informed written consent for the interview. Written informed consent will also be requested for audio recording

Theme 1 Treating undifferentiated fever

• When did you suffer fever? What did you do First action at home for treating undifferentiated fever?

• And then next step, how and where can you seek care?

• Awareness on malaria, Have you taken malaria testing? If yes, how do you take test?, who provide malaria testing? Any one suggested taking malaria testing? Who suggested to you?

Theme 2 availability, accessibility and affordability of malaria diagnostic service around the area

Where they can receive malaria testing? How many testing centers or available places around the area?

Is it easily accessible or not? Discuss on geographical, financial, easy entry point of care, clinic opening hour, communications with providers

Theme 3 Perception on diagnosis of malaria done by health care providers

• Current activities and practices

Theme 4 motivation and barriers to use malaria testing among migrant population

Motivation: Family support? Community support? Test everywhere and any when? Home care delivery service? One stop services for malaria testing and treatment? providing health education?

Barriers for not malaria testing within 24 hrs onset of fever: Lack of knowledge or information on malaria testing, No available service nearby, financial constraints, geographical and transportation constraints, providers’ level barriers?

Theme 5: Suggestions and opinions of the ways to improve the early utilization of malaria testing (what should be done by provider and what should be done by clients)

Principal investigator will complete the interview by acknowledging the time spared by the participant from his/her busy schedule. He will also give a summary of the notes taken and confirm the same from the participant.
