# Supplementary material for: Low uptake of malaria testing within 24 h of fever despite appropriate health-seeking among migrants in Myanmar: a mixed-methods study
Source: Malar J. 2018 Oct 29;17:396. doi: 10.1186/s12936-018-2546-4 (PMC6206677; doi:10.1186/s12936-018-2546-4)
Supplement: Supplementary file 4 — Additional file 4. Questions from the nation-wide migrant malaria survey 2016 that contributed to the knowledge score. [file 12936_2018_2546_MOESM4_ESM.docx]

1. Mosquito bite as the cause of malaria? Yes (1) No (0)

2. Mosquito bite transmits malaria Yes (1) No (0)

3. Whether malaria is a fatal disease? Yes (1) No (0)

4. Fever is the symptom of malaria Yes (1) No (0)

5. Chills and rigor is the symptom of malaria Yes (1) No (0)

6. Excessive sweating is the symptom of malaria Yes (1) No (0)

7. Less than five year old is a vulnerable group for malaria Yes (1) No (0)

8. Pregnant mother is a vulnerable group for malaria Yes (1) No (0)

9. Farmer is a vulnerable group for malaria Yes (1) No (0)

10. Forest related worker is a vulnerable group for malaria Yes (1) No (0)

11. Malaria is preventable Yes (1) No (0)
